# A Novel Method for Lung Image Processing Using Complex Networks

**DOI:** 10.3390/tomography8040162

**Published:** 2022-07-27

**Authors:** Laura Broască, Ana Adriana Trușculescu, Versavia Maria Ancușa, Horia Ciocârlie, Cristian-Iulian Oancea, Emil-Robert Stoicescu, Diana Luminița Manolescu

**Affiliations:** 1 Department of Computer and Information Technology, Automation and Computers Faculty, “Politehnica” University of Timișoara, Vasile Pârvan Blvd. No. 2, 300223 Timișoara, Romania; laura.broasca@cs.upt.ro (L.B.); versavia.ancusa@cs.upt.ro (V.M.A.); horia.ciocarlie@cs.upt.ro (H.C.); 2 Pulmonology Department, ‘Victor Babes’ University of Medicine and Pharmacy, Eftimie Murgu Square 2, 300041 Timișoara, Romania; oancea@umft.ro; 3 Center for Research and Innovation in Precision Medicine of Respiratory Diseases (CRIPMRD), ‘Victor Babes’, University of Medicine and Pharmacy, 300041 Timișoara, Romania; dmanolescu@umft.ro; 4 Department of Radiology and Medical Imaging, ‘Victor Babes’ University of Medicine and Pharmacy Timisoara, Eftimie Murgu Square No. 2, 300041 Timișoara, Romania; stoicescu.emil@umft.ro; 5 Research Center for Pharmaco-Toxicological Evaluations, ‘Victor Babes’ University of Medicine and Pharmacy Timisoara, Eftimie Murgu Square No. 2, 300041 Timișoara, Romania

**Keywords:** diffuse interstitial lung disease, complex networks, model, HRCT

## Abstract

The High-Resolution Computed Tomography (HRCT) detection and diagnosis of diffuse lung disease is primarily based on the recognition of a limited number of specific abnormal findings, pattern combinations or their distributions, as well as anamnesis and clinical information. Since texture recognition has a very high accuracy percentage if a complex network approach is used, this paper aims to implement such a technique customized for diffuse interstitial lung diseases (DILD). The proposed procedure translates HRCT lung imaging into complex networks by taking samples containing a secondary lobule, converting them into complex networks and analyzing them in three dimensions: emphysema, ground glass opacity, and consolidation. This method was evaluated on a 60-patient lot and the results showed a clear, quantifiable difference between healthy and affected lungs. By deconstructing the image on three pathological axes, the method offers an objective way to quantify DILD details which, so far, have only been analyzed subjectively.

## 1. Introduction

### 1.1. General Background

Pathological alterations that affect the lung interstitium usually start with an abnormally strong inflammatory process that inhibits alveoli expansion. In time, the inflammatory strain is replaced with permanent rigidity due to scar tissue, which in turn creates more inflammation, progressing towards serious clinical outcomes. This cycle of inflammation and fibrosis in the lung interstitium is the unifying characteristic of the Diffuse Interstitial Lung Diseases (DILD) group [1]. Historically, this heterogeneous group of more than 200 distinct diseases that affect the lung parenchyma have seen recurring challenges concerning the terminology, classification, and staging of the DILDs [2].

Due to having the same pathological process, the clinical and, in part, paraclinical characteristics used in DILD diagnosis tend to overlap, yet distinct pathological origins require differentiation in order to successfully issue a treatment course. There is no better example in this case than that of Idiopathic Pulmonary Fibrosis (IPF), which has a median survival rate of 2–5 years, yet its clinical diagnosis can easily be mistaken for the much more common Chronic Obstructive Pulmonary Disease (COPD) with a considerably better prognosis (mild cases have a 10–20 years survival rate) [3].

The DILD progressive aspect presents the challenge of an early and accurate diagnosis, which almost doubles the survival rate and improves life quality by employing the right treatment [4]. Clinical signs and symptoms overlap, as previously stated, so paraclinical methods are crucial to properly diagnosing DILD. However, the more commonly used investigations, like chest X-ray (CXR), peripheral blood tests, and spirometry, need to be complemented with the more specialized imagistic tools like High Resolution Computed Tomography (HRCT), lung ultrasound, and, in particular cases, bronchoscopy and surgical lung biopsy [5,6].

The HRCT has been the central non-invasive instrument in the analysis since the 2011 updated imagistic diagnostic guidelines [7], offering crucial details and insights that can lead to a swift diagnosis [8]. As with any diagnostic tool, there can be intricacies that require either a very specialized technician and/or further, more invasive, investigations. Moreover, substantial inter-observer variance, even between experienced radiologists, complicates the process [9,10,11,12]. The current approach is to try and supplement human interpretation of HRCT with automated tools, like the CALIPER software [13] or various AI-based tools [14,15,16,17].

This paper starts by briefly presenting the way diagnosis is made by computers and humans, respectively. Subsequently, a novel technique is presented and then assessed from both a biological and a system science perspective.

### 1.2. Using HRCT—Humans and Computers

In diagnosing DILDs, medical specialists start with the HRCT pattern recognition of a limited number of specific abnormal findings, particular combinations, or patterns of these abnormalities, one or more discrete distributions of abnormal findings, and the use of basic history and clinical information.

**Figure 1:**
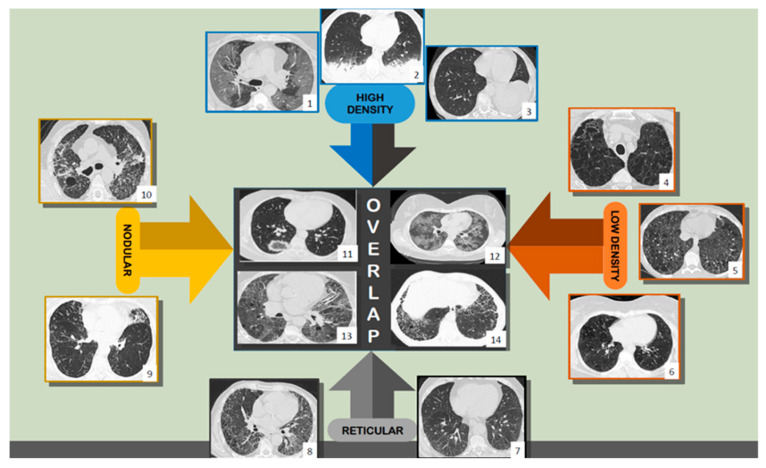
Axial thin-section CT scans, injury patterns: high density (1, 2, 3), low density (4, 5, 6), reticular (7, 8), nodular pattern (9, 10), and overlapping (11, 12, 13, 14). Scans belong to the ‘Dr. Victor Babes’ Infectious Diseases and Pneumoftiziology Clinical Hospital Timisoara database.

Radiological DILD diagnosis is pattern-based and linked to the underlying histology. It is anticipated that the future of DILD identification will involve behavior-based radiological phenotypes, with the consequence of determining clinical management [18]. By classifying primary lesion types into four categories—reticular pattern, nodular pattern, high attenuation, and low attenuation—a diagnosis can be achieved. Their overlap and association with other lesions matter [19] (Figure 1), as well as their distribution in the lung and in the basic structural and functional unit of the lung—the secondary lobule.

Thin-section CT, inspiratory, expiratory, and prone sequences comprise the most sensitive radiologic examination to evaluate the lung parenchyma for evidence of ILD. The key anatomic components of the lung parenchyma examined in IPF are the interstitium and secondary pulmonary lobule (SPL) [20]. Consequently, histological phenotypes and lesion types (primary lesions and/or its overlapping model), as well as their lung and SPL distribution, could compete and work together to indicate an accurate clinical syndrome. For example, *Usual Interstitial Pneumonia* (UIP) is the classic progressive fibrotic phenotype, yet self-sustaining progressive fibrosis is not only found in IPF patients but also in the progressive *Non-Specific Interstitial pneumonia* (NSIP) or *chronic hypersensitivity pneumonitis* (PHc) phenotype.

This (human) approach is vastly different from the way Computer Aided Diagnosis (CAD) works. Most CAD systems use heuristics and machine learning without an analytical process, being focused on proper classification and not on the underlying causes. This approach, encountered in [14,15,16,17], does not allow for any type of evaluation in terms of progress or severity. Programs that try a more anatomy-based tactic [21,22,23], commercial and scientific alike, may require additional input data, such as Pulmonary Function Tests (PFT) (e.g., Caliper), and their output only reports the abnormal volume. No qualification is offered with respect to the lesion severity in that volume. However, among the advantages of using such tools are: relatively fast processing times; verified results; good, reproducible precision; and successfully assisting the medical personnel.

The HRCT slices contain non-visual apparent information stored as Hounsfield units (HU) that can enhance the way gradient differences between pixels relate to textural differences. The various densities interweave in their geometrical placements to create textures. Since texture recognition has a very high accuracy percentage when a complex network approach is used [24,25], this paper aims to implement such a technique customized for DILD.

## 2. Materials and Methods

### 2.1. Lot Selection

To choose the eligible patients, we used ‘Dr. Victor Babes’ Infectious Diseases and Pneumoftiziology Clinical Hospital Timisoara database, stored in their private cloud archive. From more than 30000 imaging exams stored in Digital Imaging and Communications in Medicine (DICOM) format, a total of 60 scans were selected, based on the following inclusion criteria:

- 30 patients with CT exams and exploratory function tests with the diagnosis of DILD (diffuse interstitial lung disease);
- 30 patients with normal CT imaging that were considered the control group.

All the participants provided written consent for the usage of their HRCT scans. In addition, the Ethical Committee also approved this study. 

### 2.2. Imaging Parameters

All examinations were performed with a General Electric (GE) Healthcare Optima 520 16 slices with 32 slices reconstruction. The scanner is a 0.5 mm × 16 detector-row, allowing for an 8 mm total z axis length. Every patient was examined with a constant setting protocol, with variation occurring only in radiation dose due to variable tissue penetration.

The HRCT parameters used are the following:

- slice thickness: 1.25 mm;
- scan time: 1 second;
- kV: 120;
- mAs: 130;
- collimation: 2.5 mm;
- matrix size: 768 × 768;
- Field of View (FOV): 35 cm;
- reconstruction algorithm: high spatial frequency;
- window: lung window;
- patient position: supine (usually) or prone position (if DILD is suspected).

The slice is narrower than the recommended 1.5 mm by the Radiology Working Group of the Pulmonary Fibrosis Foundation to allow for better and smoother lesion detection as well as higher accuracy—both very crucial aspects of DILD diagnosis. Spatial resolution (pixel spacing) for these settings is 0.74 mm.

The HRCTs were stored in the DICOM format, as it is the universal form for encrypted medical imaging with a high transmissibility property. The algorithm behind DICOM encodes the personal information of the patients, CT information, technical parameters, and medical images, making it difficult to read without a specific application.

The main criteria for analyzing image data were the tissue densities/opacities, and these were determined by applying the Hounsfield scale’s principles. The Hounsfield units (HU) are commonly used for quantitative analysis of radio density and tissue tightness, being useful for the interpretation of CT scans. Image reconstruction relies on the tissue properties regarding X-ray beam penetration and attenuation in order to define a grayscale image system. These grayscale intervals vary between approximately −1000 HU (air) and 3000 HU (metals like silver and steel), according to the attenuation range of tissue absorption. This transformation is represented by a gray tone scale and has as a landmark the density of distilled water, which is defined as zero HU.

According to the HU intervals illustrated in Table 1, each element of this lesional picture will have an equivalent. For example, the honeycombing-pattern is a mixture of cysts (emphysema) and reticulations (consolidations); the reticular fibers’ network is a consolidation equivalent since ground-glass opacities are already represented in the table.

For the studied pathologies, the selected intervals are those representing emphysema, normal pulmonary parenchyma, ground-glass opacities, and consolidations.

### 2.3. Image Lot Selection

A 65 × 65 pixel area was manually selected out of one of the HRCT slices, for each one of the HRCT lots. The argument behind choosing such areas manually instead of processing the entire image at once is based on the idea of analyzing the most relevant samples for the chosen pathologies, taken in isolation. Only after specific patterns have been discovered would it be sensible to apply the findings on a larger scale. 

In order to remove intra- and inter- observer variability, the most relevant area for diagnosis was a majority intersection of selections made by four independent observers, two radiologists (10+ and 5+ years of thoracic experience) and two pneumologists (15+ and 5+ years of DILD experience). For the DILD-affected lot, these selections represent an extraneous diagnosis confirmation, since the images were already annotated by at least 3 specialists from the National Fibrosis Center of ‘Dr. Victor Babes’ Infectious Diseases and Pneumoftiziology Clinical Hospital Timisoara. 

The dimension for this sample area has been chosen based on multiple factors:

1. The more pixels a sample contains, the more processing power it requires to transform it into a matrix and, furthermore, into a complex network. This also influences the processing time, which could span from seconds to minutes.
2. This area should be both wide enough to capture relevant lung tissue for the diagnosis yet small enough to eliminate any extra types of tissue that might “contaminate” or add unnecessary complexity to the selected sample.
3. The selected square area should capture at least one functional component of the lung (secondary pulmonary lobule) in its entirety and, with it, any type of illness it might suffer from. Given that one secondary lobule has an area ranging from 1 cm^2^ to 2.5 cm^2^ and that the pixel spacing within the selected HRCTs varies between 0.70 and 0.80 (this setting is machine dependent and is encoded into the HRCT metadata), then a sample rectangle of 65 × 65 pixels should normally include at least one secondary lobule, e.g., actual pixel spacing value for the lot is PS = 0.74 mm, retrieved as a DICOM parameter. Given that the area of a secondary lobule is 2.5 cm^2^ × 2.5 cm^2^, then the smallest valid DICOM sample of a secondary lobule should be 25/0.74 = 33.7837 mm. However, having in mind the idea of capturing at least one full secondary lobule, the sample area size is set to almost double that value. Alternative studies have also tried similar experiments with a cropped DICOM sample of only 11 × 11 pixels, yet it is not clear why this value was chosen [22,23].

### 2.4. Image Processing Algorithm

Each of the selected samples is then processed with the help of a Python-written program developed specifically for this purpose. Using a specialized CT library, pydicom, the DICOM slices are cropped to the pre-established size (65 × 65 pixels) around the interest area.

**Figure 2:**
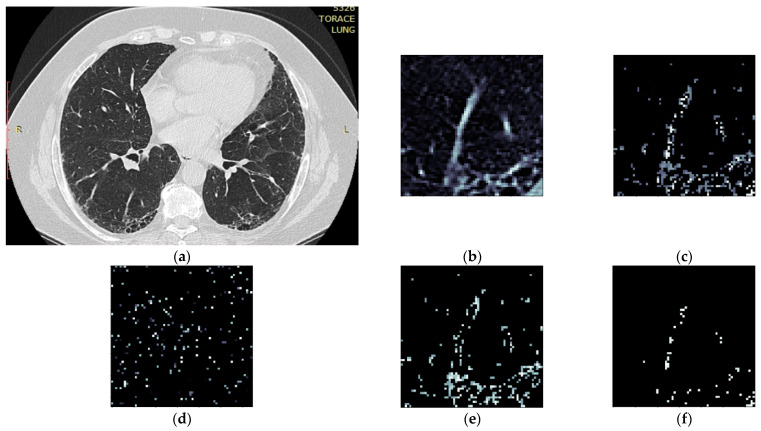
Splitting CT sample into layers (**a**) original CT, (**b**) sample crop, (**c**) combined Emphysema, GGO, and Consolidation layers, (**d**) Emphysema layer, (**e**) GGO layer, (**f**) Consolidation layer.

The program consists of an algorithm meant to carry out the following steps:

1. Iterate over a set of HRCT slices (DICOM files);
2. For each one, crop out a 65 × 65 pixel area;
3. Analyze the selected area from 3 perspectives:
  - Convert pixel gradient into a Hounsfield unit value according to the formula:
    HUv = rescaleSlope * PxGradient + rescaleIntercept,
    where rescaleSlope and rescaleIntercept are constant values dependent on the CT equipment and embedded in the DICOM metadata, and PxGradient is the color code of a pixel;
  - Isolate all emphysema-like tissue, GGO (Ground Glass Opacity), and consolidation densities in the cropped image and leave out any other types of tissue (Figure 2);
  - Separate each HU strip in the sample into a separate layer (Figure 2).
4. Generate complex networks out of each layer;
5. Analyze connectivity, closeness, and distribution of nodes (pixels);
6. Determine patterns of normal lungs and affected lungs.

In order to transform each of the crop layers (emphysema tissue, GGO tissue, and consolidation) into complex networks (Step 4), the following are assumed:

1. Each pixel represents a network node, and the pixel color (gradient) constitutes its value;
2. The two pixels are presumed to be connected if the following conditions are met:
  - The radial distance (Rd) between them (within the crop) is Rd ≤ 4 pixels. Assuming each pixel (Px) is the origin O of a circle with radius r = 4, every other pixel (Py) within the circle area can be considered connected. In other words: {∃E(Px, Py)|d(Px,Py)≤4};
  - The gradient difference between Px and Py is less than or equal to 50.

Given the above conditions, the algorithm generates sets of nodes and connecting edges, exporting them into separate files for each individual layer. Thus, each lung HU layer is converted into a complex network and analyzed from a degree distribution point of view.

Section 2.4.1 and Section 2.4.2 offer further insight into the threshold value selection processes.

#### 2.4.1. Radial Distance Selection

In order to determine the radial distance at which lesions are singular or coupled, several trials have been carried out, using values in the range 1 ≤ Rd ≤ 8 pixels. 

Values of less than 3 pixels resulted in a sparse network and very few connections, meaning that a small number of similar pixels were found in the immediate vicinity of each other. This leads to a relatively large number of scattered clusters with fewer than 3 nodes in total. When compared to other Rd values, it does not convey much relevant information about the lung profile. 

**Figure 3:**
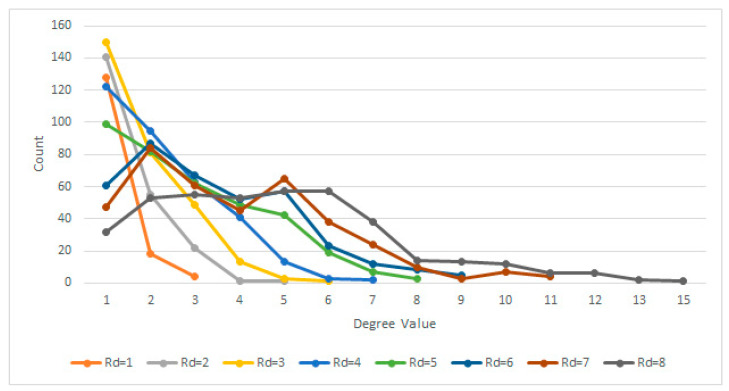
Degree distributions for various Rd.

On the other hand, with Rd values above 5, while being more integrative, the algorithm becomes too permissive due to the specific complex network process of node attachment, linking similar nodes without an anatomical cause. Defining a circle with a radius r between 5 and 8 (5 ≤ r ≤ 8) allows for a more interconnected network, fewer clusters, and a different degree distribution (Figure 3).

Therefore, given the above experiment, it has been determined based on multiple trials that the most suitable Rd value is Rd ≤ 4 pixels, which is big enough to generate dense clusters yet small enough to make a difference in terms of degree distribution, especially when comparing normal lungs with affected lungs.

This is confirmed by [29], which, in a clinical setting, uses an initial size for detectable lesions of between 3–17 mm. Since an Rd = 4 pixels corresponds to a metric value of 4 × 0.74 = 2.96 mm, the obtained empirical result matches their results. 

Further discussion regarding the distribution fit with a logarithmic or power function is presented in Section 4.1, as it refers to model fit in the network science context.

#### 2.4.2. Gradient Difference Threshold

In terms of gradient difference, the chosen delta determines whether two pixels are close enough in terms of gray tones, or if they are too far apart in terms of grayscale to be considered adjacent. While a delta D = 50 covers the entire Emphysema strip, for the GGO and Consolidation strips, it helps break the network into clusters. This rule can be summarized as:

(1) |G(Px)−G(Py)|≤D

where G(Px) and G(Py) are the respective gradient values of two pixels Px and Py, and D = 50 is the delta max threshold above which two pixels are not considered related.

In the end, each network layer can be defined as:

(2) N(P,E) where E={{Px,Py}|d(Px,Py)≤4 and |G(Px)−G(Py)|≤50}

where P is the set of vertices or pixels and E is the set of edges.

## 3. Results

**Figure 4:**
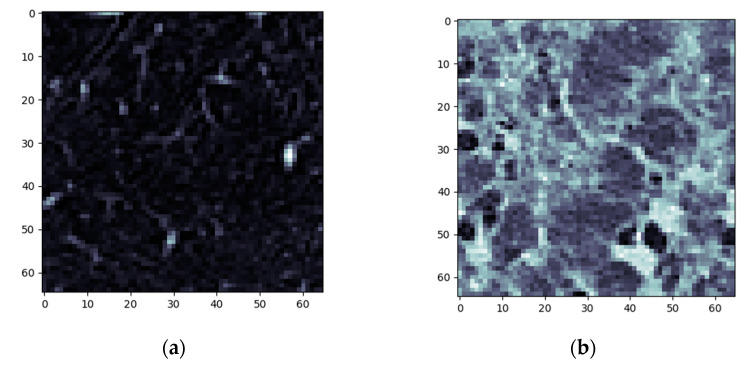
Algorithm step 1—sample selection (**a**) Normal sample (**b**) DILD (IFP) sample.

Following the previously described method, all HRCTs (of both normal and affected lungs) were processed. The individual steps for a single normal and DILD-affected patient (Figure 4) are showcased in Section 3.1, with a further lot analysis presented in Section 3.2.

### 3.1. Normal and DILD Case Sample Results

The first step is sample cropping into 65 × 65 pixels.

**Figure 5:**
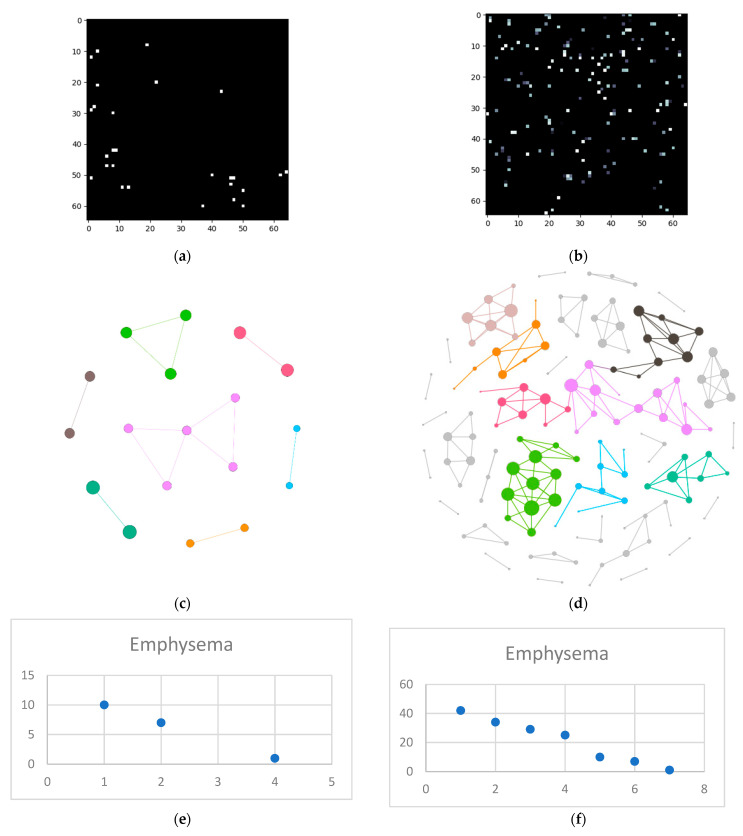
Emphysema processing (**a**) HU filtered layer for the normal sample; (**b**) HU filtered layer for the DILD sample (**c**) Complex network built according to the proposed algorithm corresponding to the normal sample, Fruchterman–Reingold render layout, node sizes proportional to node degrees, edge width invariant (1.5 pixels). (**d**) Complex network built according to the proposed algorithm corresponding to the DILD sample, Fruchterman–Reingold render layout, node sizes proportional to node degrees, edge width invariant (1.5 pixels). (**e**) Degree distribution of the normal sample network (**f**) Degree distribution of the DILD sample network.

The next steps imply splitting everything into layers and converting those layers into complex networks. First, the emphysema layer is examined (Figure 5).

**Figure 6:**
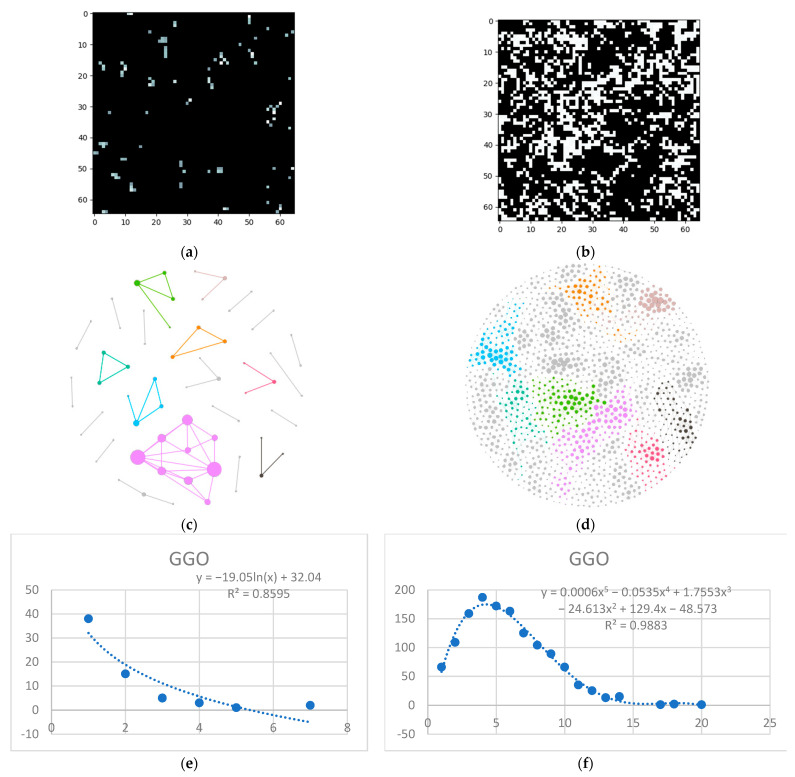
GGO processing (**a**) HU filtered layer for the normal sample; (**b**) HU filtered layer for the DILD sample (**c**) Complex network built according to the proposed algorithm corresponding to the normal sample, Fruchterman–Reingold render layout, node sizes proportional to node degrees, edge width invariant (1.5 pixels). (**d**) Complex network built according to the proposed algorithm corresponding to the DILD sample, Fruchterman–Reingold render layout, node sizes proportional to node degrees, edge width invariant (1.5 pixels). (**e**) Degree distribution of the normal sample network (**f**) Degree distribution of the DILD sample network. Equations for curve fit and R^2^ are also presented for the relevant distributions.

Next is the ground glass layer, and this is where major differences occur. Even though a visual inspection might evaluate the distributions in Figure 6a,b as random, the network degree distribution shows a completely different story: a logarithmic distribution for the normal process and a polynomial one for the IFP.

**Figure 7:**
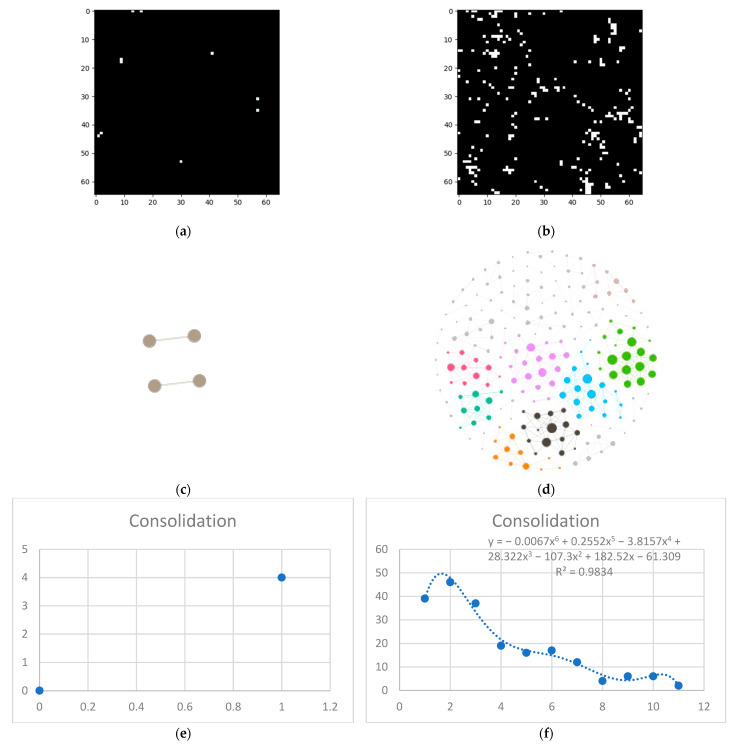
Consolidation processing (**a**) HU filtered layer for the normal sample; (**b**) HU filtered layer for the DILD sample (**c**) Complex network built according to the proposed algorithm corresponding to the normal sample, Fruchterman–Reingold render layout, node sizes proportional to node degrees, edge width invariant (1.5 pixels). (**d**) Complex network built according to the proposed algorithm corresponding to the DILD sample, Fruchterman–Reingold render layout, node sizes proportional to node degrees, edge width invariant (1.5 pixels). (**e**) Degree distribution of the normal sample network (**f**) Degree distribution of the DILD sample network. Equations for curve fit and R^2^ are also presented for the relevant distributions.

Last but not least, is the consolidation layer (Figure 7). 

### 3.2. Results 

At an individual level, the differences can be fairly distinctive, and the entire image lot analysis presented the challenge of determining network metric relevance, in a broader context. 

**Figure 8:**
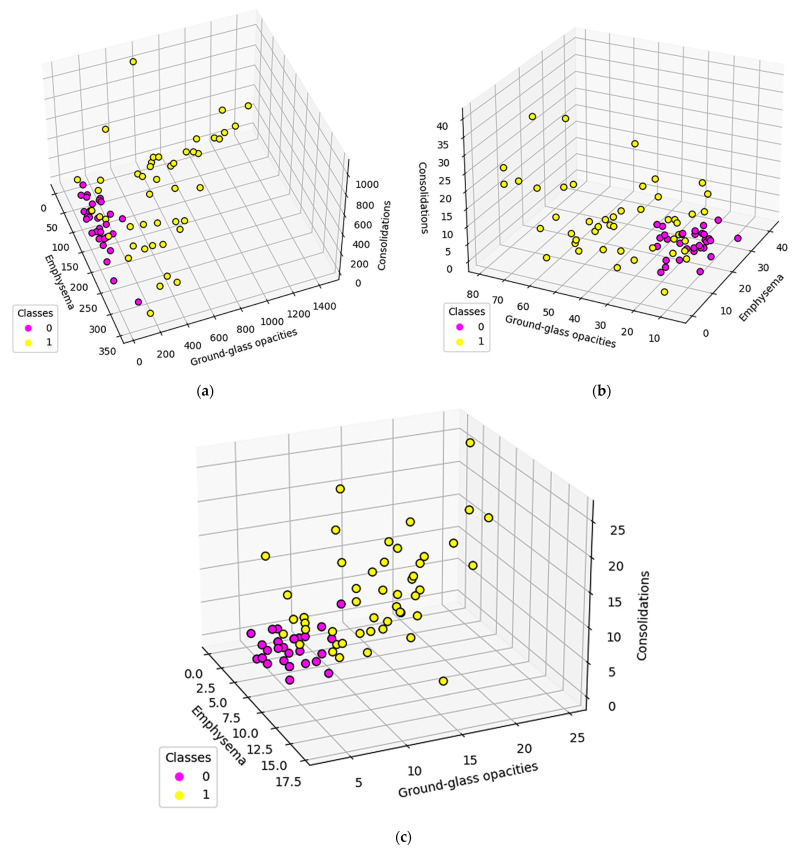
Population distribution comparisons according to specific complex network parameters: (**a**) Total count (**b**) Average count (**c**) Maximum degree Class 0 (fuchsia) represents normal lungs, while class 1 (yellow) is formed of DILD affected lungs.

In order to measure the network invariant entropy, a metric based on degree sequences is usually preferred. However, the differences shown in the previous section present the challenge of adding a measurement for the network size. Figure 8 shows three metrics, selected due to their overall balance between metrics that measure network complexity and size: total count (the degree sum), average count (average degree), and maximum degree.

**Figure 9:**
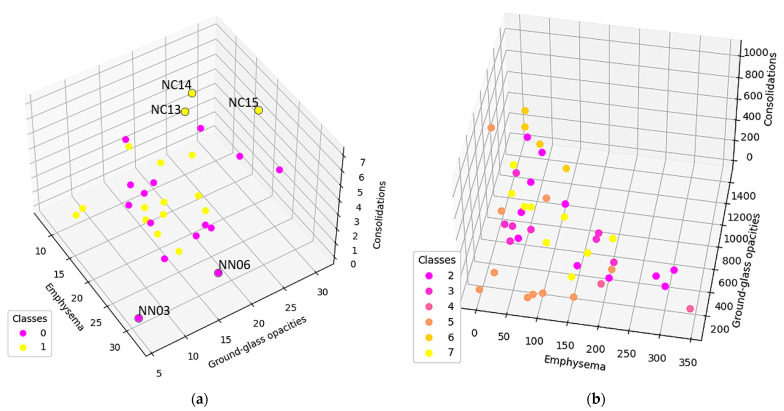
(**a**) Normal population plotted based on the average degree. Class 0 is the normal population investigated prior COVID-19, class 1 are cases diagnosed as normal in the pandemic era (**b**) DILD population plotted based on the average degree. Class 2 is UIP, 3 probable UIP, 4 UIP and emphysema, 5 organizing pneumonitis (OP), 6 hypersensitivity pneumonitis (HP), and 7 sarcoidosis.

To further study these results, normal and DILD patient distributions were plotted separately, adding another data layer (Figure 9). With normal patients, a distinction was made between patients diagnosed prior to the COVID-19 era and during COVID-19. As for DILD patients, individual disease types were highlighted.

As seen, there are a couple of outliers in what is otherwise a very tight distribution, and they will be further assessed in the discussion section.

## 4. Discussion

As stated at the beginning of the paper, the goal was to create a complex-network model based on HRCT lung imaging. Having done so, an assessment needs to be made as to how well that model fits known frameworks from network system science and medical science.

### 4.1. Network System Science

One way to describe network systems based on real-world data is through their degree distributions, more specifically by the function type best fitting those distributions. Novel research, like [30], shows that the power and logarithmic functions define these systems. Empirical results, like those presented in Figure 3, Figure 5e, Figure 6e and Figure 7e showcase a logarithmic distribution at the proper biological resolution (Rd = 4) for normal patients. However, the power function fit on all the normal patients, even varying the radius to safeguard from biological variations, is a very poor fit, especially when compared with the logarithmic function. 

**Figure 10:**
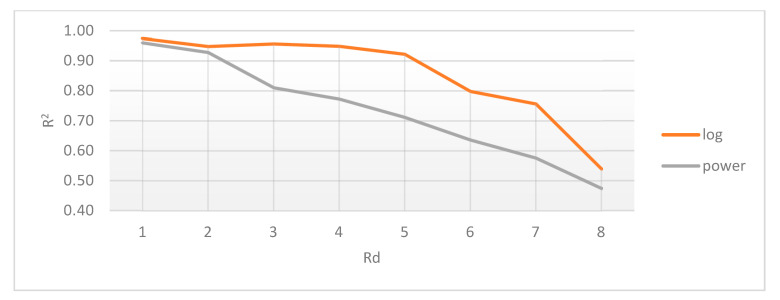
Average coefficient of determination (R^2^) for logarithmic and power distributions, relative to radial distance (Rd).

In Figure 10, the different fits of these distributions were tested against different relative distances between lung entities. Values less than 3 show a relatively similar fit, which is mathematically correct yet biologically incorrect because 1- and 2-pixel separation translate to a 0.74 mm to 1.48 mm gap, too small to be relevant.

A possible rationalization for this result comes from the perspective that biological systems with feedback have a power distribution, yet those without feedback are characterized by a logarithmic distribution, as postulated in [31]. Since the lung is a system without tightly coupled feedback loops, its distribution should follow the logarithmic model, as confirmed by our model.

Pathological lungs have an entirely different distribution, as shown in Figure 5f, Figure 6f, and Figure 7f, best fitted with a polynomial function, not a logarithmic one. Literature results show that proliferative processes have polynomial distributions [32,33] and since the studied DILDs have proliferative inflammation and fibrosis, they can be assimilated into the literature processes. Indeed, the proliferation cause is not necessarily a virus, however, the histopathological propagation still follows the same principles. 

Depending on the type of pulmonary damage, the degree of function may vary in the range of [2,8] for the studied lot. This demands further exploration with enough data to tie the degree of the polynomial function to the type or complexity of disease that a patient suffers from. To be more specific, since lung diseases manifest themselves as a composition of the three mentioned axes (emphysema, GGO, and consolidation), these three factors may vary differently in each case. So far, it can only be ascertained that there is a disease, but not its specific type. In order to be able to associate the illness complexity with a certain degree of a polynomial function, a more in-depth study, comprising separate large datasets, should be carried out.

**Figure 11:**
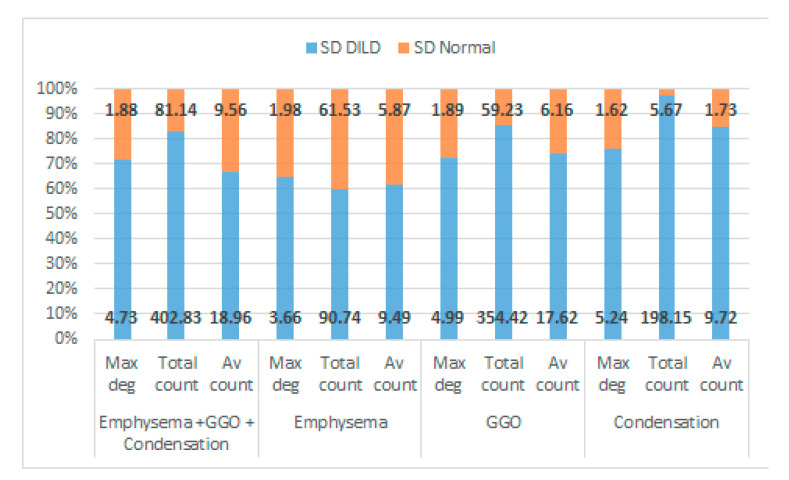
Relative percentage of standard deviation for DILD vs. normal lungs on all the pathological HU bands, taking into account maximum degree, total count, and average degree. Absolute values are also given for each data point.

The differences between the DILD-affected networks and normal networks are not only distinctive, as presented in Figure 8, but can be further quantified if a simple standard deviation for all patient data series is computed. The results, presented in Figure 11, on all the three measurements considered for the networks (maximum degree, total count, and average degree), for each HU band and for the combined pathological HU bands, prove a clear separation between the pathological and normal networks.

In conclusion, these results show that the defined model is valid from a system science perspective, accurately reflecting the underlying process that defines it.

### 4.2. Medical Science

To properly model the biological system, the presented method should reflect different anatomical and, more importantly, pathological aspects of the lung.

In Figure 9a, only the normal patients are presented, classified according to the epoch in which the exams were taken, as in pre or during the pandemic. There are three post-COVID-19 cases (NC13, NC14, and NC15) that present higher GGO and consolidation values. Studying their clinical data, NC13 and NC14 are recuperating after severe COVID-19, which would explain their artifacts. NC15, however, has a more special story, i.e., this investigation was taken before the clinical onset of COVID-19, when the PCR test result was negative. The patient went on to develop severe COVID-19, confirmed by a positive PCR test just two days later. In this case, the algorithm did detect an outlier despite the doctor’s initial diagnosis. This indicates that such an algorithm might be able to detect early changes in a patient’s lung texture and therefore offer the possibility of fast treatment if the situation warrants it. The clinical data did not show any other outliers in the NC group, as confirmed by our model.

In the pre-COVID-19 (NN) group, the outliers may occur due to patient particularities such as smokers, asthma sufferers, or convalescing post-infectious patients. For example, NN06 and NN03 (Figure 9a) are the only two heavy smokers in the normal group, whom the radiology team classified as normal. The model, however, shows them very close to the hypothetical boundary of the normal zone, reinforcing the remark that pathological and non-pathological processes are not discrete but rather a continuous process. Therefore, the granularity offered by the proposed approach enhances classical CT interpretation and offers details that could easily escape the human eye.

**Figure 12:**
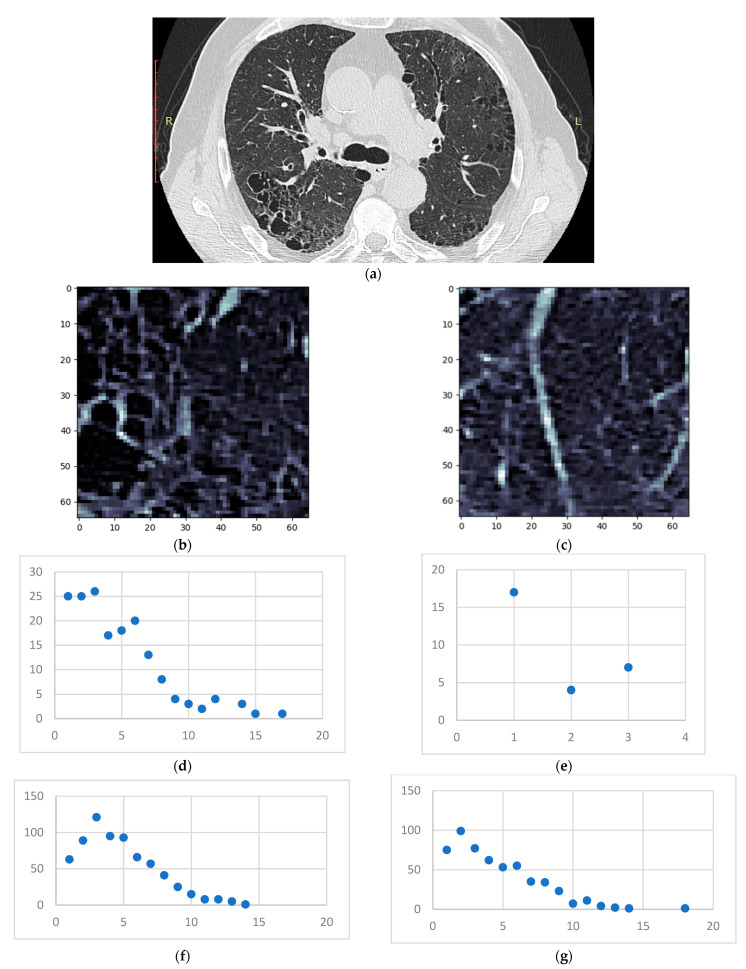
(**a**) HRCT slice under analysis (**b**) Sample 1 (**c**) Sample 2 (**d**) Degree distribution for sample 1 on the emphysema layer (**e**) Degree distribution for sample 2 on the emphysema layer (**f**) Degree distribution for sample 1 on the GGO layer (**g**) Degree distribution for sample 2 on the GGO layer.

To showcase the fitting of this model onto the pathological process, presented in the following is a case that has IPF and emphysema (Figure 12).

The emphysema bubble found in sample 1 is clearly reflected in the degree distribution of the same sample. However, both samples present similar inflammation (the GGO layer distributions), showcasing the underlying disease—IPF. The proposed model has successfully dealt with overlapping patterns in this case.

Regarding the pathological and normal case distribution presented in Figure 8, there are some cases in which the pathological points are very close to the normal ones. Zooming in, as shown in Figure 9b, those cases belong to OP (organizing pneumonitis). The OP is the usual manner of reaction to lung lesions during the healing process, consecutively most likely to a lung infection, but also found after radiation therapy, inhaling injury, neoplasm, and drug toxicity. In HRTC evaluation, it may have a multitude of appearances, including nodular images, and irregular GGO patterns, but most often peripheral bilateral consolidation (atoll sign) [34,35]. Patients that are overlapping the normal lot are (close to being) healed, therefore the model rightfully clustered them with the normal patients.

**Figure 13:**
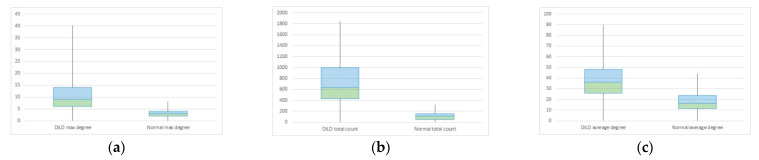
Box plot for DILD (left) vs. normal (right) for complex network parameters of (**a**) maximum degree (**b**) total count (**c**) average degree.

Comparing normal lungs with diseased lungs from a statistical perspective, is challenging due to different DILD phenotypes and the relatively small lot size/disease class. To prove the method and model work in an overall manner, a t-test: two-sample assuming unequal variances was conducted, comparing normal to DILD samples. The results, summarized in Table 2 and Figure 13, show that measured p is less than 0.05 (3.97 × 10^−17^, 8.52 × 10^−23^, and 5.31 × 10^−9^) and observed t (10.49, 14.91, and 6.29) is larger than critical t (1.98, 1.99, and 1.98), therefore rejecting the null hypothesis; i.e., being 95% confident that the differences between groups are not due to chance.

Proper comparisons between disease phenotypes would require a much larger study in order to be relevant. However, the purpose of this paper was to test if the complex network model can accurately reflect the biological process and the quantitative data agrees. From a qualitative medical science perspective, the matter needs further study, yet the results seem encouraging.

### 4.3. Comparisons with other HRCT Analysis Methods

In this section, this method is compared with existing ones. Assessed against the normal, established way of analyzing the HRCT by human radiologists and doctors, the proposed method is almost too simple. The full medical analysis is not limited to the HRCT; it will almost always require clinical data and, more often than not, other paraclinical investigations. Regarding the modality, human analysis uses a difficult-to-reproduce mixture of analytical and empirical processes (“clinical sense”) and its disease progress measurement is mostly subjective [9,10,11,12]. 

CAD methods vary from commercial to research ones. The most well-known commercial approach, Caliper [21], does not use just HRCT; it also needs a way to calculate lung expansion, like PFT. However, it is a very objective, stand-alone way to measure lung diseases and works remarkably fast. The proposed method is not nearly as fast as it needs an estimated median of 2 min/sample for the whole three layers, therefore requiring 242 min/full slice and 3872 min/patient. The time values are measured on an average PC running a single-threaded program. Amdahl’s law indicates that there is room for improvement, with some limitations. This is a downfall and needs work in order to reach full analysis capacity, although the information offered is multifaceted compared with Caliper, due to the complex network methodology.

Research-stemmed approaches, like the one from [22,23] and any of the ones based on machine learning like [14,15,16,17], use just the HRCT, but the way they offer measurement for the disease is inexistent in most cases and volumetric in others. Most machine learning approaches are oriented towards proper classification and pattern identification and not as a way to quantify it. Also, the time aspect is mostly unspecified for all these approaches, so no assessment can be made.

A summary of all these comparisons is offered in Table 3.

None of the aforementioned approaches offers a way to mathematically characterize affected areas of the lung, unlike the present method. Using network characteristics, it can quantify and qualify a pathological process on three axes. However, it is still unable to work alone and needs many more cases to allow for proper classification methods.

## 5. Conclusions

In this paper, a novel method of using complex networks to transform lung HRCT has been presented. The methodology section delves deeper into the algorithm steps and the justification of each chosen parameter. The sample size is justified by anatomical bounds of the secondary pulmonary lobule; the radius influencing network connectivity is correlated with injury granularity and the Hounsfield unit intervals are dependent upon the device and resolution. The results section presents in parallel the processing steps for two sample patients (a normal and a pathological one), as well as a whole-lot perspective. In the discussion section, the correctness of this model is justified from a system science perspective, by using the degree distributions as the main system characterization tool. Furthermore, the network measurement clusterization is described, showing that it results in clear disparities between the normal and pathological lots. From a medical science perspective, it is showcased how the chosen model reflects clinical data and how its low granularity presents an advantage in the diagnosis process. In the end, a comparison between this method and other existing ones highlights the advantage that it has: to offer a complex qualitative and quantitative measurement. Pitfalls of the proposed model include its inability to work alone yet and the relatively small lot on which it was tested, which will all need to be addressed in further research.

In conclusion, the stated goal is considered to have been achieved, by showing how a complex network model can be used to transmute lung HRCT into a quantifiable and qualifiable structure that can enhance the DILD diagnosis.

## Figures and Tables

**Table 1:** HU intervals from the reports of Lin Li et al. and Maria Paola Belfiore et al. [26,27,28]. These values are specific to the General Electric Healthcare Optima 520.

Pulmonary Zones	HU Intervals
Emphysema	[−1024, −977)
Normal pulmonary parenchyma	[−977, −703)
Ground-glass opacities	[−703, −368)
Others (crazy-paving, pleural fat)	[−368, −100)
Consolidations	[−100, 5)
Others (interstitial vessels)	>5 HU

**Table 2:** Statistical comparisons.

	Maximum Degree		Total Count		Average Count	
	DILD	Normal	DILD	Normal	DILD	Normal
Mean	15.96875	7.032258	846.5692	7.1	51.65253	32.53397
Variance	39.45933	3.365591	206,084.5	3.334483	362.9068	113.4483
Observations	30	30	30	30	30	30
Hypothesized Mean Difference	0		0		0	
Df	82		64		92	
t Stat	10.49451		14.9084		6.288591	
P(T ≤ t) one-tail	3.97 × 10^−17^		8.52 × 10^−23^		5.31 × 10^−9^	
t Critical one-tail	1.663649		1.669013		1.661585	
P(T ≤ t) two-tail	7.93 × 10^−17^		1.7 × 10^−22^		1.06 × 10^−8^	
t Critical two-tail	1.989319		1.99773		1.986086	

**Table 3:** Methodology compariso.ns.

	Just HRCT	Analytical	Empirical	Works Alone	Measurement
Doctor	N	Y	Y (“clinical sense”)	Y	Subjective
Caliper [21]	N, PFT	Y	N	Y	Yes, one dimensional size
Zrimec [22,23]	Y	Y	N	Mostly	Maybe
Machine learning	Y	N	Y	Maybe	Maybe
Proposed model	**Y**	**Y**	**N**	**N**	**Yes, three dimensional size**

## Data Availability

The data presented in this study are available on reasonable request from the corresponding author.

## Abbreviations

CAD	Computer Aided Diagnosis
COPD	Chronic Obstructive Pulmonary Disease
CT	Computer Tomography
DILD	Diffuse interstitial lung Diseases
FOV	Field of View
GE	General Electric
GGO	Ground Glass Opacity
HRCT	High Resolution Computed Tomography
HPc	Chronic Hypersensitivity Pneumonitis
HU	Hounsfield Unit
ILD	Interstitial Lung Diseases
IPF	Idiopathic Pulmonary Fibrosis
NSIP	Non-Specific Interstitial Pneumonia
OP	Organizing Pneumonitis
PCR	Polymerase Chain Reaction
PFT	Pulmonary Function Test
SPL	Secondary Pulmonary Lobule
UIP	Usual Interstitial Pneumonia

## References

[1] Ryu J.H., Daniels C.E., Hartman T.E., Yi E.S. (2007). Diagnosis of Interstitial Lung Diseases. Mayo Clin. Proc..

[2] Guler S.A., Corte T.J. (2021). Interstitial Lung Disease in 2020: A History of Progress. Clin. Chest Med..

[3] Molina-Molina M., Aburto M., Acosta O., Ancochea J., Rodríguez-Portal J.A., Sauleda J., Lines C., Xaubet A. (2018). Importance of early diagnosis and treatment in idiopathic pulmonary fibrosis. Expert Rev. Respir. Med..

[4] Kolb M., Richeldi L., Behr J., Maher T.M., Tang W., Stowasser S., Hallmann C., du Bois R.M. (2017). Nintedanib in patients with idiopathic pulmonary fibrosis and preserved lung volume. Thorax.

[5] Meyer K.C. (2014). Diagnosis and management of interstitial lung disease. Transl. Respir. Med..

[6] Manolescu D., Davidescu L., Traila D., Oancea C., Tudorache V. (2018). The reliability of lung ultrasound in assessment of idiopathic pulmonary fibrosis. Clin. Interv. Aging.

[7] Sverzellati N. (2013). Highlights of HRCT imaging in IPF. Respir. Res..

[8] de Bois R.M. (2012). An earlier and more confident diagnosis of idiopathic pulmonary fibrosis. Eur. Respir. Rev. Off. J. Eur. Respir. Soc..

[9] Inomata M., Jo T., Kuse N., Awano N., Tone M., Yoshimura H., Moriya A., Bae Y., Terada Y., Furuhata Y. (2021). Clinical impact of the radiological indeterminate for usual interstitial pneumonia pattern on the diagnosis of idiopathic pulmonary fibrosis. Respir. Investig..

[10] Walsh S.L.F., Calandriello L., Sverzellati N., Wells A.U., Hansell D.M., UIP Observer Consort (2016). Interobserver agreement for the ATS/ERS/JRS/ALAT criteria for a UIP pattern on CT. Thorax.

[11] Walsh S.L.F., Wells A.U., Desai S.R., Poletti V., Piciucchi S., Dubini A., Nunes H., Valeyre D., Brillet P.Y., Kambouchner M. (2016). Multicentre evaluation of multidisciplinary team meeting agreement on diagnosis in diffuse parenchymal lung disease: A case-cohort study. Lancet Respir. Med..

[12] Trusculescu A.A., Manolescu D., Tudorache E., Oancea C. (2020). Deep learning in interstitial lung disease-how long until daily practice. Eur. Radiol..

[13] Crews M.S., Bartholmai B.J., Adegunsoye A., Oldham J.M., Montner S.M., Karwoski R.A., Husain A.N., Vij R., Noth I., Strek M.E. (2020). Automated CT Analysis of Major Forms of Interstitial Lung Disease. J. Clin. Med..

[14] Simonyan K., Zisserman A. (2015). Very Deep Convolutional Networks for Large-Scale Image Recognition. arXiv.

[15] Li Q., Cai W., Wang X., Zhou Y., Feng D.D., Chen M. Medical image classification with convolutional neural network. Proceedings of the 13th International Conference on Control Automation Robotics Vision (ICARCV).

[16] Walsh S.L.F., Calandriello L., Silva M., Sverzellati N. (2018). Deep learning for classifying fibrotic lung disease on high-resolution computed tomography: A case-cohort study. Lancet Respir. Med..

[17] Li Q., Cai W., Feng D.D. Lung image patch classification with automatic feature learning. Proceedings of the 2013 35th Annual International Conference of the IEEE Engineering in Medicine and Biology Society (EMBC).

[18] Walsh S.L.F., Kolb M. (2018). Radiological diagnosis of interstitial lung disease: Is it all about pattern recognition?. Eur. Respir. J..

[19] Signs and Patterns of Lung Disease—Chest Radiology: The Essentials.

[20] Hobbs S., Chung J.H., Leb J., Kaproth-Joslin K., Lynch D.A. (2021). Practical Imaging Interpretation in Patients Suspected of Having Idiopathic Pulmonary Fibrosis: Official Recommendations from the Radiology Working Group of the Pulmonary Fibrosis Foundation. Radiol. Cardiothorac. Imaging.

[21] Chen A., Karwoski R.A., Gierada D.S., Bartholmai B.J., Koo C.W. (2020). Quantitative CT Analysis of Diffuse Lung Disease. RadioGraphics.

[22] Zrimec T., Busayarat S. (2011). Computer-aided Analysis and Interpretation of HRCT Images of the Lung. Theory and Applications of CT Imaging and Analysis.

[23] Depeursinge A., Zrimec T., Busayarat S., Müller H. 3D Lung Image Retrieval Using Localized Features. Proceedings of the Medical Imaging 2011: Computer-Aided Diagnosis.

[24] de Lima G.V.L., Castilho T.R., Bugatti P.H., Saito P.T.M., Lopes F.M. (2015). A Complex Network-Based Approach to the Analysis and Classification of Images. Progress in Pattern Recognition, Image Analysis, Computer Vision, and Applications.

[25] Mourchid Y., Hassouni M.E., Cherifi H. (2019). A General Framework for Complex Network-Based Image Segmentation. Multimed. Tools Appl..

[26] Li L., Qin L., Xu Z., Yin Y., Wang X., Kong B., Bai J., Lu Y., Fang Z., Song Q. (2020). Using Artificial Intelligence to Detect COVID-19 and Community-acquired Pneumonia Based on Pulmonary CT: Evaluation of the Diagnostic Accuracy. Radiology.

[27] Belfiore M.P., Urraro F., Grassi R., Giacobbe G., Patelli G., Cappabianca S., Reginelli A. (2020). Artificial intelligence to codify lung CT in COVID-19 patients. Radiol. Med..

[28] Grassi R., Belfiore M.P., Montanelli A., Patelli G., Urraro F., Giacobbe G., Fusco R., Granata V., Petrillo A., Sacco P. (2021). COVID-19 pneumonia: Computer-aided quantification of healthy lung parenchyma, emphysema, ground glass and consolidation on chest computed tomography (CT). Radiol. Med..

[29] Hiramatsu M., Inagaki T., Inagaki T., Matsui Y., Satoh Y., Okumura S., Ishikawa Y., Miyaoka E., Nakagawa K. (2008). Pulmonary ground-glass opacity (GGO) lesions-large size and a history of lung cancer are risk factors for growth. J. Thorac. Oncol. Off. Publ. Int. Assoc. Study Lung Cancer.

[30] Smith K.M. (2021). Explaining the emergence of complex networks through log-normal fitness in a Euclidean node similarity space. Sci. Rep..

[31] Adler M., Mayo A., Alon U. (2014). Logarithmic and Power Law Input-Output Relations in Sensory Systems with Fold-Change Detection. PLoS Comput. Biol..

[32] Shang Y. (2015). Degree distribution dynamics for disease spreading with individual awareness. J. Syst. Sci. Complex..

[33] Pastor-Satorras R., Castellano C., Van Mieghem P., Vespignani A. (2015). Epidemic processes in complex networks. Rev. Mod. Phys..

[34] Hovinga M., Sprengers R., Kauczor H.-U., Schaefer-Prokop C. (2016). CT Imaging of Interstitial Lung Diseases. Multidetector-Row CT Thorax.

[35] Desai S.R., Prosch H., Galvin J.R., Hodler J., Kubik-Huch R.A., von Schulthess G.K. (2019). Plain Film and HRCT Diagnosis of Interstitial Lung Disease. Diseases of the Chest, Breast, Heart and Vessels 2019-2022: Diagnostic and Interventional Imaging.

